# A cluster randomised controlled trial in primary dental care based intervention to improve professional performance on routine oral examinations and the management of asymptomatic impacted third molars: study protocol

**DOI:** 10.1186/1748-5908-2-12

**Published:** 2007-04-20

**Authors:** Theodorus G Mettes, Wil JM van der Sanden, Michel Wensing, Richard PTM Grol, Alphons JM Plasschaert

**Affiliations:** 1 Radboud University Nijmegen Medical Centre, Department of Preventive and Restorative Dentistry, College of Oral Sciences, Nijmegen, The Netherlands; 2 Radboud University Nijmegen Medical Centre, Centre for Quality-of-care Research (WOK), Nijmegen, The Netherlands

## Abstract

**Background:**

Routine oral examination (ROE) refers to periodic monitoring of the general and oral health status of patients. In most developed Western countries a decreasing prevalence of oral diseases underpins the need for a more individualised approach in assigning individualised recall intervals for regular attendees instead of systematic fixed intervals. From a quality-of-care perspective, the effectiveness of the widespread prophylactic removal of mandibular impacted asymptomatic third molars (MIM) in adolescents and adults is also questionable. Data on the effectiveness of appropriate interventions to tackle such problems, and for promoting continuing professional development in oral health care are rare.

**Methods/design:**

This study is a cluster randomised controlled trial with groups of GDPs as the unit of randomisation. The aim is to determine the effectiveness and efficiency of small group quality improvement on professional decision-making of general dental practitioners (GDPs) in daily practice. Six peer groups ('IQual-groups') shall be randomised either to the intervention arm I or arm II. Groups of GDPs allocated to either of these arms act as each other's control group. An IQual peer group consists of eight to ten GDPs who meet in monthly structured sessions scheduled for discussion on practice-related topics. GDPs in both trial arms receive recently developed evidence-based clinical practice guidelines (CPG) on ROE or MIM. The implementation strategy consists of one interactive IQual group meeting of two to three hours. In addition, both groups of GDPs receive feedback on personal and group characteristics, and are invited to make use of web-based patient risk vignettes for further individual training on risk assessment policy. Reminders (flow charts) will be sent by mail several weeks after the meeting.

The main outcome measure for the ROE intervention arm is the use and appropriateness of individualised risk assessment in assigning recall intervals, and for the MIM-intervention group the use and appropriateness of individualised mandibular impacted third molar risk management. Both groups act as each other's control. Pre-intervention data will be collected in study months one through three. Post-intervention data collection will be performed after nine months.

## Background

Routine oral examination (ROE) refers to periodic monitoring of the general and oral health status of patients. The main purpose of ROEs is to prevent the onset of oral diseases and/or prevent further progression. This allows the introduction of preventive interventions at the appropriate time, and reduces the need for operative interventions. In most developed Western countries, a decreasing prevalence of oral diseases underpins the need for a more individualised approach in assigning individualised recall intervals for regular attendees instead of systematic decision-making of fixed intervals. In The Netherlands, about 80% of the population regularly visits the dentist for a check-up about every six months [1]. This implies that many healthy individuals are scheduled for routine oral screening. In 2000, 50% of the Dutch GDPs assigned all their regular patients for ROE twice a year [2], irrespective of level of risk for oral disease. The efficiency of this systematic monitoring system is still disputed in The Netherlands, as well as internationally [3-10]. Recently, two systematic reviews [11,12] and a clinical practice guideline (CPG) advocated an individualised risk-based assessment strategy, given the lack of good scientific evidence [13]. In addition to the debate over the frequency of ROE, GDPs also question from a quality-of-care perspective the effectiveness of the widespread prophylactic removal of mandibular impacted asymptomatic third molars (MIM) in adolescents and adults [14-16].

Recent implementation studies in medical care indicate that evidence on the effect of single interventions is mixed [18,19]. It is as yet unclear how quality of oral care in dental practice can be improved. Research data on effectiveness of interventions to promote continuing professional development for dentists are rare [17]. A previous study showed that small group education sessions did not change dentists' clinical behaviour [20]. The aim of the present study is to evaluate whether a multifaceted strategy can enhance oral health care according to evidence-based dental practice. Consensus-based CPGs on ROEs [13] and on the management of MIMs [20] are available for educational purposes in clinical practice.

### Aim of the study

To determine the effectiveness and efficiency of small group quality improvement on professional decision-making of general dental practitioners (GDPs) concerning risk assessment in ROEs (including assigned recall intervals) and risk management of MIMs for patients (children and adults) in dental practice.

### Scientific hypothesis

Multifaceted implementation of consensus-based clinical practice guidelines (CPGs) for GDPs on ROEs and the management of MIMs in daily dental practice is more effective and efficient compared to dissemination of CPGs only.

## Methods

### Study design

The study is a cluster-randomised trial with incomplete block design. In one trial arm, the intervention focuses on individual decision-making in scheduling ROEs. In the second arm, the intervention focuses on monitoring and decision-making regarding prophylactic removal versus retention of MIM. Groups of GDPs allocated to either of these arms act as each other's control group. To reduce potential contamination, groups of GDPs are randomised rather than individual GDPs (Table 1). We assumed that the two clinical conditions (or practices) were largely independent of one another, i.e. performing one would not necessarily influence the other. In the ROE arm, the CPG only mentions the necessity of third molars screening in general as routine oral care. In the MIM arm, the CPG provides an extensive, but specific, decision-making algorithm, i.e. how to deal with mandibular asymptomatic impacted third molars.

**Table 1:** Balanced incomplete block design

**Intervention**	**CPG**	
	**ROE**	**MIM**

Group I (ROE)	Intervention	Control
Group II (MIM)	Control	Intervention

Intervention: Clinical Practice Guideline (CPG) on the management of routine oral examinations (ROE) and asymptomatic mandibular impacted third molars (MIM).

### Recruitment of GDPs and inclusion/exclusion criteria

Dental peer groups ("IQual-group"), each comprising at least eight participating GDPs, are the unit of randomisation. An IQual group consists of GDPs who attend monthly sessions scheduled for discussion on practice-related topics as part of a quality assurance program. Participants in peer groups generally support quality-improvement procedures, and are experienced in continuing dental education and professional cooperation. The Dutch Dental Association (NMT) has initiated this system, and supports nationwide dental peer groups extensively, e.g., offering professional support, feedback and continuing education programmes. All IQual-groups were invited to participate in this study by a general announcement on the NMT website, dependent on their ability to begin the study within two to three months. Those groups that were interested in participating were invited to visit a section of the NMT website  for members only that provided more detailed information on the project.

### GDP inclusion criteria

The inclusion criteria consisted of:

• GDPs who work for at least for three days a week in general dental practice for a minimum of three years

• GDPs who have a patient population of regular ROE-attendees and manage their patient records electronically.

• GDPs were required to give their informed consent for the assessment and evaluation of electronic patient records. Patient data are collected anonymously.

### Patient's inclusion criteria

To be eligible for inclusion in the study, all patients must have regularly visited the same dentist at least once a year for ROEs over the preceding three years. For the MIM arm, patients should also be between 17 and 35 years of age, and with disease-free impacted mandibular third molars in retention.

### Patient's exclusion criteria

For the ROE arm, patients with symptomatic-driven (emergency) attendance in dental practice, or regular attendance in the participating dental practice of less than three years, are excluded from the study. For the MIM arm, patients with symptomatic or previously removed third molars, or regular attendance in the participating dental practice of less than three years, are excluded from the study

## Intervention

### Implementation strategy

Participants in both trial arms receive a recently developed evidence-based CPG on ROE or MIM. The implementation strategy consists of one interactive IQual group meeting of approximately two to three hours with a minimum of eight GDPs each. These meetings discuss the selected intervention topic, and offer a more risk-based decision-making process guided by the CPG. Topics regarding risk management, such as identification of risk factors/indicators, preventive interventions, prognosis, monitoring, record keeping, and patient scheduling are presented. In addition, all participants receive feedback from personal and group characteristics retrieved from pre-test questionnaire and specific record forms, and are invited to make use of web-based patient risk vignettes for further individual training on risk assessment policy. These risk vignettes were developed by structured consensus procedures (modified Delphi) with expert groups consisted of acknowledged GDPs and oral surgeons in special fields. In addition, reminders (flow charts) and written patient leaflets with topical information are provided during the trial period. Flow charts comprise algorithms of decision-making aspects linked to the trial arm allocation. Depending on the allocated trial arm, participants are subjected to a set of planned interventions as described in Table 2.

**Table 2:** Overview of planned interventions in groups I and II.

**Interventions for all IQualgroups**		
Composition IQual group Introductory letter (individual) Delivery registration forms and questionnaires	Questionnaire GDPs 25 observations chair side	

**Randomisation**		

**Interventions trial arms**	**ROE group I**	**MIM group II**

Delivery CPG on ROE versus MIM by post	CPG ROE	CPG MIM
Education session IQual group	ROE education	MIM education
Online training website (individual feed back)	Access to ROE-based training	Access to MIM-based training
Reminder (flow chart), individual feed back record form Feed back by email	ROE- aspects Flow chart	MIM-aspects Flow chart
Registration in practice (25)	25 observations in practice chair side	25 observations in practice chair side
End trial	Questionnaire	Questionnaire

### Randomisation

After their commitment to participate, 60 GDPs nested in six IQual groups were randomly assigned (using SPSS) as groups to the ROE or MIM arm by an independent secretary not familiar with the groups. The unit of randomisation was the IQUAL group.

### Outcomes and instruments

#### ROE study

Table 3 lists the outcome parameters and instruments used. For the ROE arm, the primary outcome measure is the use and appropriateness of individualised risk assessment measured through the assigned recall intervals (in months). The appropriateness will be assessed as follows:

**Table 3:** Outcome parameters and instruments

**Outcome parameter**		**Instruments**
**Primary ROE-outcomes**	Clinical Performance/decision-making: Number of patients per GDP with assigned recall interval (months) based on individual risk profile assessment. For high-risk children and adolescents' intervals less than seven months, in case of low risk profile more than seven months; for low-risk adults' profiles, nine months equal or more, and for high-risk adults' profiles less than nine months.	**Patient record, registration form, to analyse risk management**
**Secondary ROE-outcomes**	Clinical Performance/decision-making: Number of patients per GDP with prescribed individual frequency of BWs (months). For high-risk children and adolescents, frequencies of less than 24 months, and for low-risk profiles, frequencies of more than 36 months; for high-risk adults, prescription frequencies less than 36 months, and for low risk adults, prescription of more than 48 months. Number of patients per GDP with periodontal DPSI-score > 1, and prevalent caries, who have been given feedback, information and preventive advice, registered in patient record or registration form. Efficacy data/cost-effectiveness scores: Mean overall length in months of recall intervals per GDP over the past 3 yrs Mean total number of BW(s) and other radiographs over past 3 years Type of performer GDP/Oral hygienist/others (level of graduation, education) Total number of additional interventions performed during ROE (polishing, removal of calculus: coded as M50, M55). Professional attitudes and compliance: Measured at the beginning and end of the trial, by questionnaire.	**Patient record, registration form, questionnaire to analyse additional performance and cost-analysis**
**Primary MIM-outcome**	Clinical performance/decision-making: Number of patients (between 17 -35 yr of age) with removed versus retained MIMs in accordance with CPG, or with indication for removal. Number of risk-based assessment radiographs between 17– 35-yrs/per patient with risk-based for assessment of prognosis MIM.	**Patient record, registration form to analyse risk management**
**Secondary MIM-outcome**	Professional attitudes/compliance and feedback: Interviews of patients (17–35 years of age) to confirm risk-based performance.	**Questionnaire**

• For high-risk children and adolescents (0 to 18 years), recall intervals of less or equal than seven months should be assigned. For those with a low-risk profile, an assigned recall of more than seven months is considered appropriate.

• For high-risk adults (18 years and older): recall intervals of less than nine months should be assigned. For those with a low-risk profile, an interval of nine months or longer is considered appropriate.

The secondary outcome measures for the ROE arm are:

1. The use and appropriateness of individualised risk-based assessment in prescribing bitewing radiographs (BWs) in months. The appropriateness will be assessed as follows:

• For high caries-risk children and adolescents (0 to 18 years): BW frequencies of less than 24 months are determined as appropriate; for those with a low-caries risk profile, BW frequencies equal or more than 36 months.

• For high caries risk adults (18 years and older): BW frequencies less than 36 months are determined as appropriate; for those with a low-caries risk profile, BW frequencies of equal or more than 48 months.

2. The use and appropriateness of individualised communication/feedback and advice in patients with a periodontal risk DPSI-score >1, and present dental caries experience. The appropriateness will be assessed as the proportion of patients per GDP receiving appropriate preventive advice/feedback will be calculated. Furthermore, as a secondary outcome measure, professional role perceptions and compliance concerning the recommendations of the ROE-CPG is assessed by means of questionnaires provided at the beginning and end of the study.

3. Resource use will be documented for an economic evaluation:

• The type of recall interval (months) per GDP over the past 3 years

• BW radiographs and other types of radiographs per GDP over the past 3 years

• Type of performer of ROEs: GDP versus oral hygienist/dental auxiliary

• Additional interventions per GDP (i.e. polishing stains/removing dental calculus) encompassed at ROEs over the past 3 years.

#### MIM study

For the MIM arm, the primary outcome is the use and appropriateness of individualised MIM risk management. The appropriateness will be assessed as follows:

• Patients (17–35 years of age) with removed versus retained MIMs over the past five years as a proportion of patients aged between 17–35 years of age per practice

• Radiographs used for monitoring patients mentioned above to perform a risk-based assessment and prognosis of MIM over the past five years.

A secondary outcome measure is GDPs- attitudes and compliance concerning the recommendations of the MIM-CPG, and relating that information to patients. This measure will use data from patient interviews to confirm risk-based performance.

All data will be collected using special registration forms to be completed by GDPs and patient records available in practices. Questionnaires, patients' records, and registration forms will provide information to assess all outcome parameters. The structured registration forms were used in a previous self-recording study [23].

### Data collection

After their informed consent to participate, GDPs will be invited to first complete a questionnaire to collect personal and practice characteristics, as well as aspects of attitude and compliance. Individual assessment of electronic patient records with regard to the outcome measures, combined with a special registration form (to be applied individually in daily practice), will be used during the evaluation period.

Baseline information will be collected before randomisation of groups, as well as at the end of the trial after seven to nine months. Each GDP will be instructed to complete at least 20 forms per registration period. As each peer group consists of at least eight participants, and each arm will consist of three groups, this will result in a minimum of 480 registrations per trial arm. Finally, questionnaires will be collected from GDPs, dentist's assistants and co-workers to assess acceptance and applicability.

### Sample size

The primary outcome measures in this study are collected from individual patients who are clustered within GDPs. GDPs are clustered within (existing) IQual groups which have been randomised to one of the two arms of the trial. The power calculation assumes that the primary outcomes are dichotomous measures, although some outcomes might be treated as continuous measures as well. On the basis of previous research and experience with IQual groups, we expect a relatively high clustering of scores within GDPs, for instance, the intra-cluster coefficient (ICC) for recall interval assignment was 0.29 [23], and a low clustering of scores within IQUAL groups (changing professional behaviour is largely determined by other factors). We use the ICC for clustering in IQual groups, because this was the unit of randomisation. We aim for a 20% change on primary outcomes (e.g. 20 to 40% patients receive individualised recall intervals). Assuming *a power of *80%, alpha = 0.05 and an effect size of 20% for both interventions and an estimated ICC of 0.03 based on previous estimates [21,22], the (Aberdeen) power calculation [24] revealed that six IQual groups (60 GDPs) should comprise 150 registrations (patients) per group, resulting in at least 450 registrations in each trial arm.

### Statistical analysis

The primary analysis will be performed on an intention-to-treat-analysis. Secondly, measures will be constructed in particular algorithms to define the appropriateness in variables. Thirdly, the impact on each of the primary and secondary outcomes will be estimated separately, using random effects regression models (linear or logistic) to take into account the clustering of data. These basic models include group allocation (intervention, control), measurement timing (baseline, post-intervention), and interaction of group allocation and measurement timing (=intervention effect). Fourthly, prognostic factors for the outcome (which may be confounders) will be added to the models, like patients' recall interval preferences, which varies from those assigned by GDPs, as well as the preferences regarding the prescription of radiographs by patients/GDPs. In addition, this also accounts for GDPs and patients' preferences regarding removal versus retention of asymptomatic impacted third molars. Fifthly, a limited number of subgroup analyses will be performed, including an analysis of effectiveness in participants which performed all activities as planned, i.e., education session, online training program, and helpdesk (= efficacy analysis).

## Economic evaluation

An economic evaluation is performed to estimate the cost-effectiveness of the implementation intervention. This study takes a healthcare perspective and a time horizon that is similar to the implementation trial.

### Effectiveness

The effects are defined in terms of professional performance, because measuring health outcomes or health utilities is beyond the scope of the study. Outcome measures will be the same as in the trial (e.g. oral health risks assessment performance and guideline adherence regarding individual recall assignment and individual monitoring of impacted asymptomatic third molars) and extracted from the trial data.

### Costs

Costs considered are those used for the implementation (time for participation by GDPs, preparation time, use of materials) and for changes (if any) in professional performance (recall intervals between successive ROEs, total number of radiographs, both based on individual risk assessment). Oral care unrelated to the topic of the interventions within the observed time period will not be considered. Resource use will be extracted from trial data, where possible, or collected separately for the purpose of the economic evaluation. Costs will be valued according to prevailing Dutch guidelines for economic evaluations, and alternatively according to the current national fee-coding list for individual oral treatment procedures in general dental practice.

### Analysis

An incremental cost effectiveness ratio (ICER) will be constructed that expresses the ratio of differences of costs and effects between the study arms (for each of the two clinical topics). Uncertainty will first be examined in one-way sensitivity analyses of the most influential factors. Finally, a non-parametric bootstrap re-sampling analysis will be performed, which provides a cost-effectiveness plane for a simulated sample of 1,000 drawings (with put-back) from the pool of observed cost-effect pairs.

These data will be compiled from questionnaires, patient risk profiles, registration forms, and from electronic patient records. All instruments were pre-tested in a pilot study. Measurements and analysis of pre-test data will be performed before or during the intervention period (for retrospective data sampling), and after the intervention period (post-intervention data).

### Timeframe of the study

We plan to randomise six of the initially recruited IQual groups that have declared their willingness to participate in this study, and to assign them randomly to one of the two intervention arms. The baseline data collection will take place at the beginning of the study during months one and two. The intervention will start in months two and three, and follow-up data collection will be collected in months eight through ten. The scheduled time for the trial is estimated to be seven to ten months (Appendix 1), assuming that each GDP will collect data from at least 20 regular attending patients by means of the trial registration form.

## Discussion

Little evidence was available on the estimates of the likely size of dental primary care ICCs, and which prognostic factors influenced their magnitude. Based on research in this field, we assumed a substantial variation in primary dental care between fairly autonomous GDPs [25-28]. Data extracted from primary health care suggested that ICCs for patient outcomes in primary care were generally less than 0.05 [21,22]. In reviews of this protocol, questions were raised about the power calculation. In particular, the expected effect size was seen as large, and the applied ICC as low. This would imply that the power calculation is too optimistic, and that the study might be underpowered to detect meaningful change in professional behaviour.

### Ethical and legal aspects

The study protocol was approved by the Ethics Committee of the Radboud University Nijmegen Medical Centre, prior to the start of the study in September 2006 (approval number CMO nr. 2006/168). All patient data and other confidential information fall under dental confidentially rules, and are stored on a protected server of the Radboud University Nijmegen Medical Centre. Only members of the study team have access to the files.

## Authors' contributions

All authors declare that they have no competing interests. DM, WvdS and MW performed the study and drafted the manuscript. AP and RG participated in the study design. All authors have read and approved the final manuscript.

## Source of funding

This study is funded by a research grant from The Health Care Insurance Board (CVZ) in Amsterdam The Netherlands.

**Figure 1:**
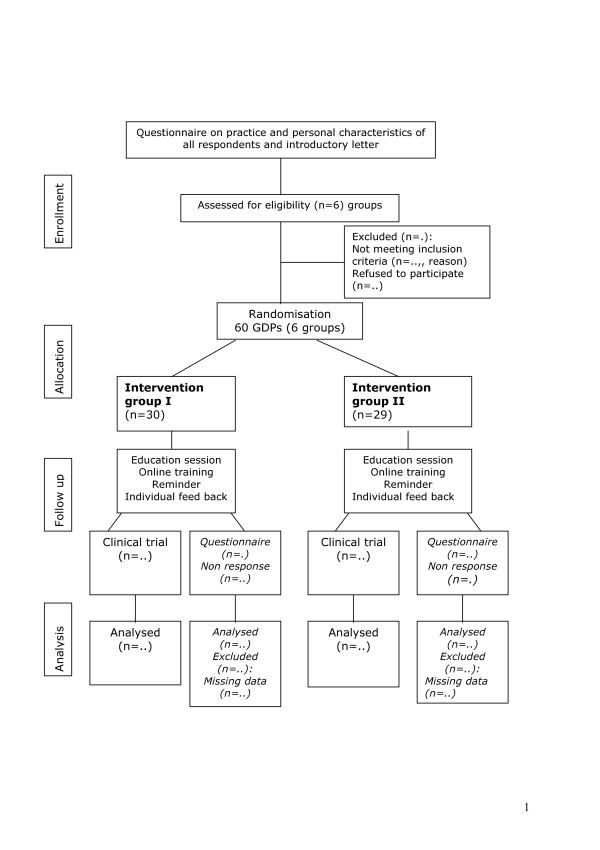
Design timeframe implementation study concerning CPGs on ROE and MIM.

**Figure 2:**
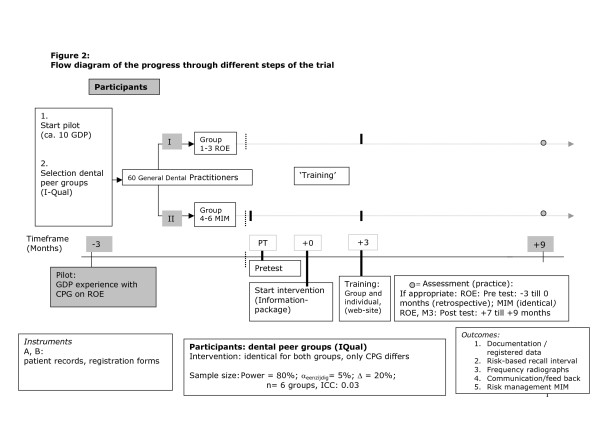
Flow diagram of the progress through different steps of the trial.
